# Personal Values and Innovative Behavior of Employees

**DOI:** 10.3389/fpsyg.2019.00865

**Published:** 2019-04-18

**Authors:** Ewelina Purc, Mariola Laguna

**Affiliations:** The John Paul II Catholic University of Lublin, Lublin, Poland

**Keywords:** values, innovation, innovative behavior, job autonomy, employees

## Abstract

Innovations are based on the good ideas of individuals; therefore, it is very important to better understand the role that individuals and their personal characteristics play in innovative initiatives. The aim of the current study was to test the relationships between employees’ personal values and their innovative behavior. It was hypothesized that these relationships are mediated by an employee’s job autonomy. We integrated Schwartz’s basic human values theory with the notion that job autonomy is an important job characteristic that can be redesigned to better fit employees’ preferences. The study results (obtained from 263 employees in different branches) showed that openness to change and self-enhancement values are positively related to job autonomy, whereas conservation and self-transcendence values are negatively related to job autonomy, which confirms that personal values are important in explaining autonomy in the workplace. In addition, employees’ self-enhancement values are positively related to their innovative behavior, while conservation and self-transcendence values are negatively related to innovative behavior. Mediation analysis with a bias-corrected bootstrapping method showed that job autonomy is a significant mediator of the relationships between employees’ personal values (except for openness to change) and their innovative behavior. Our research extends the theory of basic human values, showing that values serve as a personal basis for innovative behavior. Our results also contribute to the innovation research by demonstrating the importance of personal values and job autonomy for innovative behavior in organizations.

## Introduction

Innovation is widely recognized as important for the effectiveness and success of organizations (Yuan and Woodman, 2010; Anderson et al., 2014; Razmus and Laguna, 2018). Due to the growing demands and expectations of customers and the global expansion of markets, innovation has become important for companies (Anderson et al., 2018). The importance of innovation has also been noted by scientists, and research concerning innovation and creativity has garnered much attention from scholars in the last 20–30 years (de Jong and den Hartog, 2010). Although there is a significant amount of empirical evidence regarding the antecedents of innovative behavior in organizations, there is still a need for more research on predictors (Hammond et al., 2011). It is particularly important to better recognize the psychological mechanisms that are conducive to employee innovation, extending the knowledge gained from management research investigating organizational variables. In their recent review, Anderson et al. (2018) called for more research to broaden our understanding of individual innovation in organizations. Addressing this gap in the literature, we explain employees’ innovative behavior in our study.

Employees are the individuals who create and implement innovative solutions in organizations; therefore, their behaviors are critical to organizational innovation. The literature provides evidence of some individual innovation antecedents (for a review, see West, 2002; Anderson et al., 2004, 2014, 2018; Hammond et al., 2011); however, only recently has research started to investigate the role of personal values in explaining innovation. Because personal values are the guiding principles in people’s lives, affecting their goals and actions (Schwartz, 1992), it is important to study their roles in employees’ innovation (Anderson et al., 2014). It is particularly promising because values are postulated as being important drivers of actions in organizational settings (Meglino and Ravlin, 1998; Sagiv et al., 2011a). However, empirical studies concerning these relationships are scarce.

Responding to this literature gap, the present study applies Schwartz’s theory of basic human values (Schwartz, 1992) to explain which factors foster innovative behavior in employees. We also postulate the potential mechanism, testing job autonomy as a mediator in the relationships between personal values and innovative behavior. In addition, we propose a new approach to job autonomy as an individual perception of a workplace setting that can be fostered by an employee’s personal values. In the subsequent sections, detailed explanations concerning all relationships that are considered in this study will be presented.

The study contributes to the literature by providing new insight into Schwartz’s theory of basic human values (Schwartz, 1992), job characteristics theory (Hackman and Oldham, 1976), and the innovation literature. Namely, it extends these theories by testing whether personal values motivate people to shape their work conditions and stimulate their innovative behavior in the workplace. Moreover, whereas most of the previous research has focused on the organizational level of innovation (see meta-analyses: Damanpour, 1991; Rosenbusch et al., 2011), our research proposes a conceptual model of a mechanism stimulating employees’ workplace innovation, combining both individual and contextual factors. Based on this approach, we answer the recent call in the innovation literature to reveal the mechanisms through which innovation can be driven (Hammond et al., 2011; Anderson et al., 2014, 2018).

### Relationships Between Employees’ Personal Values and Innovative Behavior

Innovation, defined as the generation or adoption of useful and novel ideas that are effectively introduced in organizations (Amabile, 1988; Verhees and Meulenberg, 2004; Rosenbusch et al., 2011), is important for their business success (Rosenbusch et al., 2011). Innovation in organizations includes the introduction not only of big ideas that significantly change existing practices but also of small, incremental improvements in coping with daily challenges at work (Amabile, 1988; Camisón-Zornoza et al., 2004; Weinberger et al., 2018). The small-scale innovations manifesting themselves in everyday innovative behavior are based on creative ideas (Weinberger et al., 2018). However, innovative behavior includes not only generating ideas (which is specific for creativity; Amabile, 1988) but also implementing them in organizations (Scott and Bruce, 1994). As creativity is considered a first step toward innovation (West and Farr, 1992; Amabile, 1996; Anderson et al., 2014), in the subsequent sections, we utilize both the creativity and innovation literature to build our arguments and hypotheses concerning the relationship between employees’ personal values and innovative behavior.

The theory of basic human values proposed by Schwartz (1992) is currently considered to be the most comprehensive and empirically grounded approach to human values (Sagiv et al., 2011a; Cieciuch, 2013). Schwartz argued that values are “desirable transsituational goals, varying in importance, that serve as guiding principles in the life of a person or other social entity” (Schwartz, 1994, p. 21). Values have motivational power by providing direction and emotional intensity to action and by being acquired through socialization, in the context of dominant group values, as well as through individual learning (Schwartz, 1994). The central assumption of the theory is that basic values form a universal, circular continuum and are organized in accordance with the motivation that they express. Relationships between motivations can be compatible, conflictual, or irrelevant to one another (Schwartz, 1992). Due to its structure, the value continuum can be partitioned in different manners (Sagiv and Roccas, 2017). Ten initially described basic values can be structured into the following two bipolar dimensions: (1) openness to change (self-direction and stimulation) versus conservation (tradition, conformity, and security) and (2) self-transcendence (universalism and benevolence) versus self-enhancement (power and achievement); hedonism values share aspects of both dimensions (Schwartz, 1992). A distinction between the four higher-order values representing the endpoints of these two dimensions (i.e., openness to change, conservation, self-enhancement, and self-transcendence) is frequently used in research (e.g., Vecchione et al., 2015; Cieciuch et al., 2016) and will be applied in our study.

Personal values are closely related to motivation and thus help explain behavior (Cieciuch, 2017). Behavior, which expresses people’s individual values, enables them to attain their goals and personal aspirations (Sagiv and Schwartz, 2000; Bardi and Schwartz, 2003). People are motivated to behave in accordance with their values because they look for a sense of consistency between their beliefs and actions (Rokeach, 1973). Employees are therefore willing to rely on their personal values in making decisions, choosing actions, and justifying their behavior (Arieli and Tenne-Gazit, 2017). The inability to implement and realize individual values in the workplace has been found to be positively related to job burnout (Retowski and Podsiadły, 2016) and negatively related to job satisfaction (Amos and Weathington, 2008).

Personal values, being guiding principles in life, can also affect people’s creativity and innovative behavior (Anderson et al., 2014). Indeed, some studies have shown such relationships (Rice, 2006; Dollinger et al., 2007; Kasof et al., 2007; Lipponen et al., 2008; Sousa and Coelho, 2011). Nevertheless, this evidence is relatively scarce and is partially derived from student samples (e.g., Dollinger et al., 2007; Kasof et al., 2007). Therefore, there is a need to systematically examine how exactly personal values are related to innovative behavior in the workplace, a point that has been recently emphasized by scholars (Anderson et al., 2014, 2018). Bardi and Schwartz (2003), p. 5, stated that “the natural way to pursue important values is to behave in ways that express them or promote their attainment.” Therefore, we expect some values to foster innovative behavior in employees and others to be negatively related to it. As very little empirical research investigating such relationships has been conducted, our hypotheses are based mostly on theoretical assumptions derived from Schwartz’s values theory (Schwartz, 1992) and on research findings concerning creativity.

The higher-order value of openness to change comprises self-direction and stimulation (Schwartz, 1992). According to the theory of basic human values, the motivational goals of openness to change are a willingness to choose, create, and explore and a preference for novelty (Schwartz, 1992) and change (Ros et al., 1999). Self-direction has been argued to be the value that is the most important for creativity for at least two reasons (Dollinger et al., 2007). First, creativity was one of the specific values used by Schwartz (1992) to capture self-direction. Second, because the motivational goal of self-direction involves independence in thought and action, self-direction can be reflected through exploration and free choice in following individual interests, which are perceived to be crucial for creative individuals (Helson, 1990; Barron, 1997). Because self-directed people prefer to be independent both in thought and in action, this value seems to be conducive not only to the generation of creative ideas but also to innovation implementation. The motivational goal of stimulation in Schwartz’s theory (Schwartz, 1992) involves seeking novelty, excitement and challenges in life. Therefore, the value of stimulation also seems to promote innovative behavior as a method of attaining these goals. These characteristics allow us to suppose that openness to change values will be the most favorable to innovative behavior among all other higher-order values. Indeed, researchers have reasoned that due to their motivational meanings, openness to change values are associated with innovation and creativity (Arieli and Tenne-Gazit, 2017), and previous empirical research has confirmed the relationships between these constructs. Kasof et al. (2007) found that both self-direction and stimulation are positively related to individual creative performance. Another study showed that openness to change values positively predict creativity (Dollinger et al., 2007). Employees who ranked low on openness to change were found to be less creative (Sousa and Coelho, 2011), and self-direction was positively related to employee creative behavior (Rice, 2006). Based on Schwartz’s (1992) theory, we can expect that when employees strongly value novelty, experimentation, and exploration (typical for people with openness to change values), they will be willing to behave innovatively. Because of these theoretical assumptions and previous research findings, we developed the following hypothesis.

*Hypothesis 1a*. Employees’ openness to change values are positively related to their innovative behavior.

In contrast, conservation values, which include conformity, security, and tradition (Schwartz, 1992), seem to have a negative effect on employees’ innovativeness. Such values predispose an individual to accept customary behavior and established procedures and ideas, which are undoubtedly not conducive to innovativeness (Schwartz and Bardi, 2001; Schwartz, 2006; Sousa et al., 2012). Because the motivational goal of conformity is to restrain actions, inclinations, and impulses to avoid upsetting or violating social norms and expectations (Schwartz, 1992), employees who attribute high importance to this value may avoid undertaking innovative initiatives because it may produce changes that are not easily welcomed by others in their organizations. Employees’ security values also do not seem to be favorable for innovative behavior because they focus on stability, safety, and harmony, whereas implementing innovations in companies often requires breaking the status quo and disrupting established organizational conventions, norms, and procedures. Tradition values emphasize the acceptance of imposed, traditional customs and ideas (Schwartz, 1992). Innovative activities are not congruent with such an approach, and employees who want their innovative ideas to be implemented in organizations should definitely take the initiative on their own and strive for idea realization, which sometimes requires substantial effort. These theoretical expectations are somewhat supported by previous research findings. Dollinger et al. (2007) confirmed the negative relationship between conservation values and creative accomplishments. A study conducted by Lipponen et al. (2008) revealed that employees who emphasized conservation versus openness to change values suggested fewer new initiatives for change in the workplace. In addition, employees who ranked high on conservation values tended to be less creative than those who ranked high on openness to change values (Sousa and Coelho, 2011), and employees who prioritized conformity were less creative than those who instead preferred self-direction (Rice, 2006). Moreover, Kasof et al. (2007) found that all three conservation components – tradition, conformity, and security – were negatively related to creative performance. Based on these premises, we expect that an employee who is not willing to introduce novelty and rejects alternative, unfamiliar methods and new perspectives (which is typical for those who hold conservation values) will not be willing to behave innovatively (including idea generation, promotion and implementation) because it may potentially disturb the status quo. Thus, we developed the following hypothesis.

*Hypothesis 1b.* Employees’ conservation values are negatively related to their innovative behavior.

Self-enhancement values are reflected in power and achievement (Schwartz, 1992), both of which focus on social esteem. Power reflects the goals of prestige, social status attainment, and control or dominance over people and resources. Implementing innovative ideas in the workplace can be a potential method of attaining such goals because employees who behave innovatively can be appreciated by managers who strive for innovative performance at their firms (Janssen et al., 2004). An employee can also perceive innovative behavior as a means to obtain social prestige in an organization and to have a leading, distinguished position among others. Furthermore, the central goal of the value of achievement is personal success, which is accomplished by demonstrating competence, in accordance with social standards (Schwartz, 1992). Innovative activities can help to achieve such a goal because an innovative employee may attain a distinguished position among co-workers and can be perceived as being successful. An employee’s innovative behavior may also be appreciated by supervisors, leading to benefits such as financial bonuses or promotions, which may indicate prestige and status. Nevertheless, previous research findings concerning relationships between self-enhancement values and creativity are not consistent. On the one hand, Dollinger et al. (2007) found that power values had a negative effect on creativity. On the other hand, Sousa and Coelho (2011) found that bank employees who attributed high importance to self-enhancement values were more creative in their work. In addition, Taştan and Davoudi (2017) demonstrated that both power and achievement values had a positive effect on organizational innovativeness among employees in managerial positions. These results seem to correspond with the finding that power motivation is important for creative personality (Helson, 1996), and the notion that strong achievement orientation is demonstrated by creative people (Mumford, 2000; Sousa and Coelho, 2011). Attaining goals related to power and achievement values may be possible when people promote and implement their creative ideas. These activities can help employees gain prestige, increase their organizational status, and be perceived as successful by co-workers and supervisors. In conclusion, we postulate that employees who attribute high importance to self-enhancement values are more willing to behave innovatively.

*Hypothesis 1c.* Employees’ self-enhancement values are positively related to their innovative behavior.

Self-transcendence values consist of universalism and benevolence (Schwartz, 1992). They reflect an individual’s basic need to establish social relations with other people (Arieli and Tenne-Gazit, 2017). Being the most abstract among values, they seem to be the most unrelated to the work context (Sousa et al., 2012). However, there is some empirical evidence showing that they can be related to creativity. Gump (2007) found that universalism positively predicts creativity among college students. Similarly, Kasof et al. (2007) showed that universalism is positively correlated with undergraduate students’ creative performance. In the study conducted by Dollinger et al. (2007), higher self-transcendence values predicted both higher creative accomplishments and more creative products. Nevertheless, these studies were conducted using student samples. Conversely, Sousa and Coelho (2011) found that frontline bank employees who attributed high importance to self-transcendence were less creative than those who had stronger self-enhancement values. Although there is some empirical evidence concerning the relationship between self-transcendence and creativity, we do not consider it to be sufficient to postulate a specific hypothesis about how these values are related to innovative behavior in the workplace as an activity that includes idea generation, promotion, and implementation. The lack of sufficient evidence is due to some inconsistencies in previous research and – above all – the lack of clear theoretical premises on the potential relationship direction between these variables.

### Relationships Between Employees’ Personal Values and Autonomy

Personal values are considered to be the core of personality, affecting attitudes, evaluations, and decisions (Feather, 1988) and acting as a guiding force to peoples’ perceptions and actions (Schwartz, 1994). Therefore, values can also be related to employees’ job autonomy. We propose that a specific set of values can predispose people to proactively strive for autonomy in their work, while other values may not motivate such a pursuit.

Autonomy is known to be one of the most frequently studied phenomena in work and organizational settings (Morgeson and Humphrey, 2006). It is a motivational tool (Sarros et al., 2002; Biron and Bamberger, 2010) leading to positive work outcomes, such as innovation and creativity (Hammond et al., 2011; Liu et al., 2011; De Spiegelaere et al., 2014), job satisfaction, internal work motivation (see Humphrey et al., 2007) and work engagement (Halbesleben, 2010). In most of these studies, autonomy is conceptualized, following Hackman and Oldham’s (1976) job characteristics theory, which classifies autonomy as one of the core job characteristics and defines it as the degree of freedom and independence provided by a job. Such freedom can be reflected in making decisions, scheduling work, and determining work methods and procedures applied in an organization. Another meaningful theoretical approach is self-determination theory (Ryan and Deci, 2000), which considers autonomy as one of the three basic psychological needs and suggests that the satisfaction of these needs is necessary for people to flourish (Deci and Ryan, 2000). In this context, autonomy is known to be supported by supervisors and their human resource practices (Park and Jang, 2015), whereas in job characteristics theory (Hackman and Oldham, 1976, 1980), autonomy is acknowledged as an objective task characteristic that can also be provided by the job itself. To integrate these approaches and to extend them using insights from new theories explaining employees’ proactive functioning (Wrzesniewski and Dutton, 2001; Tims and Bakker, 2010), we propose another perspective to capture employee job autonomy. We suggest that (1) job autonomy is, to some extent, dependent on the work environment and supervisor actions, such as human resource practices, as postulated by the job characteristics theory (Hackman and Oldham, 1976, 1980); however, (2) to some extent, job autonomy can also be shaped by the employee on his/her own. This argument aligns with the conception of proactive actions as “the physical and cognitive changes individuals make in the task or relational boundaries of their work” (Wrzesniewski and Dutton, 2001, p. 179). Employees make such self-initiated changes in their job features to customize them to fit their strengths, passions, and motives (Berg et al., 2008). Traditional job design theories, such as job characteristics theory (Hackman and Oldham, 1976), consider managers as job crafters because they design tasks for their subordinates and, therefore, can change their motivations and satisfaction (Wrzesniewski and Dutton, 2001). However, employees are able to proactively redesign their jobs on their own, and such self-initiated changes made in an employee’s own job demands and job resources are postulated to help them attain or optimize their work goals (Tims et al., 2012). Indeed, research has shown that employees who participated in job redesign initiatives experienced increases in job autonomy after 2 months (Tims et al., 2013). Therefore, there is support for the theoretical postulates that job autonomy can be influenced not only by managers through top-down processes but also by employees on their own. In this manner, we define job autonomy by integrating existing theoretical conceptions.

As noted by Morgeson and Humphrey (2006), autonomy has a central place in motivational work approaches. In addition to being the most widely studied job characteristic, it is also the most influential (Humphrey and Morgeson, 2008). Moreover, job autonomy is the job characteristic related to innovative behavior (e.g., Liu et al., 2011; De Spiegelaere et al., 2014; Orth and Volmer, 2017), and it also seems to be related to personal values. Thus, we concentrate on job autonomy in our study.

We assume that an individual can strive to have more autonomy at work when it is congruent with his/her personal values. As personal values have been proven to develop in the early stages of life and then be relatively stable across time (Vecchione et al., 2015, 2016; Cieciuch et al., 2016), and as job autonomy is more likely to change in relation to the organizational context, the job itself, and the relationship between the supervisor and the subordinate (Hackman and Oldham, 1976, 1980), we treat values as predictors of job autonomy. Based on Schwartz’s (1992) theory, we expect that employees can be more or less disposed toward seeking autonomy in their work based on the basic personal values they prefer. A person can be highly motivated to have an opportunity to make decisions and feel independent at work because it is of central significance to him/her, while another person might focus on other attributes of the job and not strive for autonomy because he/she does not consider it to be important for his/her work functioning. As noted by Sagiv and Roccas (2017), p. 4, values “represent desirable goals and reflect what people consider important and worthy.” For instance, when an employee attributes high importance to openness to change values, which focuses on autonomy in thought and action, novelty, and challenge, we can expect that he/she will pursue the highest possible job autonomy. However, when an employee emphasizes conservation values, the core of which is to maintain the status quo and to follow norms and rules, he/she will be not as motivated to strive for autonomy at work. Certainly, the fact that a job is autonomous is also, to some extent, determined by other factors, such as the nature of the job itself (e.g., artistic professions will be naturally more autonomous than receptionist or cashier jobs), or by managers, who may or may not allow their subordinates to make decisions, schedule their work, or choose work methods on their own. Nevertheless, drawing on the basic human values theory (Schwartz, 1992), we expect job autonomy to be predicted by employees’ personal values. Below, we formulate hypotheses related to each of the four higher-order values.

Schwartz’s (1992) theory characterizes openness to change values, which includes self-direction and stimulation, as being focused on “independent action, thought and feeling, and readiness for new experience” (Schwartz, 2003a, p. 269). The central goal of self-direction is the person’s independence, both in thinking and in acting. Schwartz states that self-direction is based on the organismic needs for, on the one hand, control and mastery and, on the other hand, requirements of autonomy and independence (Schwartz, 1992). Stimulation is described as being focused on novelty, challenge, and excitement. This value type is derived from a need for stimulation and variety to maintain an optimal level of activation (Schwartz, 1992). These theoretical assumptions concerning self-direction and stimulation, which constitute the openness to change values, suppose that these values are particularly conducive to job autonomy in employees. Sagiv and Schwartz (2004) argued that among career counseling clients, self-direction is relevant to initiating actions, self-reliance, and independence of thought in making career decisions. Indeed, their findings confirmed that the priority clients gave to self-direction was positively correlated with their independent behavior, as assessed by career counselors. There is also some evidence concerning the role of personal values in professional choice. A stronger emphasis on openness to change values predicts artistic and investigative careers (Sagiv, 2002; Knafo and Sagiv, 2004) and entrepreneurial career intentions (Gorgievski et al., 2017). Based on these theoretical and empirical premises, we expect that the importance that employees attribute to openness to change values is positively related to their work autonomy.

*Hypothesis 2a.* Employees’ openness to change values are positively related to their job autonomy.

Conservation values, which include the values of conformity, security, and tradition, are in conflict with openness to change. Conformity values are focused on self-restraint, including self-restraint of actions, impulses, and inclinations, which are reflected in everyday interactions with close others (Schwartz, 1992). Valuing security motivates the maintenance of harmony, stability, and safety of the self and relationships with others and society. The tradition value emphasizes the respect and acceptance of imposed traditional ideas and customs. Together, the conservation values encourage status quo maintenance, resistance to change and self-restriction to avoid violating social norms (Schwartz, 2003a). These characteristics do not seem to be conducive to pursuing autonomy in the workplace. An employee who attributes high importance to conservation values may accept the existing situation and not strive to enhance his/her job autonomy because it can be harmful for organizational rules and norms. He/she may be afraid that attempts to increase work autonomy could be negatively perceived by superiors or other co-workers. There is some empirical evidence that can shed some light on the potential relationship direction between an employee’s conservation values and his/her work autonomy. In a study conducted by Sagiv and Schwartz (2004), clients’ emphasis on conformity values was found to be negatively related to their independent behavior, which they expressed in the career counseling process. Moreover, an emphasis on conservation values predisposes individuals to engage in rather conventional professions, such as accountants, administrative managers, or receptionists, and to hold vocational interests that demand following well-defined instructions and rules, systematic operations, and obeying norms (Sagiv, 2002; Knafo and Sagiv, 2004). Because peoples’ professional choices affect the types of behavior in which they are willing to engage in the workplace (Holland, 1997; Arieli and Tenne-Gazit, 2017), we expect that people who attribute great importance to conservation values do not strive to enhance their job autonomy because it is not congruent with their values. Therefore, we developed the following hypothesis.

*Hypothesis 2b.* Employees’ conservation values are negatively related to their job autonomy.

The higher-order value of self-enhancement includes power and achievement (Schwartz, 1992). Power is focused on attaining prestige, social status, dominant position, and control over people and resources. The value of achievement is concentrated on personal success, which can be attained through competence demonstration. The theoretical assumption is that employees who attribute high importance to self-enhancement values will strive to have more autonomy in their workplaces. Feeling autonomous and independent at work seems to be crucial to attaining dominance and control over other co-workers and to developing self-interest goals. An employee who is self-confident and autonomous can express his/her competence in the workplace. Thus, the motivational goals of self-enhancement can be attained. A highly autonomous job is more challenging and creates feelings of personal responsibility and control of outcomes at work (Hackman and Oldham, 1980; Sousa et al., 2012). Mumford (2000) argued that power and achievement are strong motives for people who tend to be independent. In addition, career counseling clients’ achievement values were found to be positively related to their independent behavior (rated by counselors) (Sagiv and Schwartz, 2004), and higher self-enhancement values predicted entrepreneurial career intentions in students from different countries (Gorgievski et al., 2017). Based on these premises, we expect that employees’ self-enhancement values are positively related to their autonomy at work.

*Hypothesis 2c.* Employees’ self-enhancement values are positively related to their job autonomy.

The higher-order value of self-transcendence encompasses universalism and benevolence. Universalism is focused on the welfare of all people, as well as nature (Schwartz, 1992). The motivational goal of benevolence involves concern for people who are relatively close, and this concern is expressed in everyday interactions. As previously stated, self-transcendence is the most abstract higher-order value, and it has been argued that it is not as strongly related to work context as other values (Sousa et al., 2012). Nevertheless, there is some empirical evidence concerning the effects of the self-transcendence values on work-related issues. These values were found to be positively related to altruistic and pro-social behaviors at work, in contrast to the self-enhancement values (Sosik et al., 2009; Schwartz, 2010). Moreover, in a study that used social dilemma games, Sagiv et al. (2011b) found that the participants who attributed high importance to self-transcendence were more willing to cooperate with others than those who emphasized self-enhancement. However, there is no empirical evidence on the relationship between self-transcendence and work autonomy. Based on theoretical assumptions, we can expect that striving for autonomy is not highly important to employees who emphasize self-transcendence. Instead, these employees are likely focused on cooperating with co-workers, showing their concern for others and being tolerant of all people. Nevertheless, theoretical and empirical evidence does not seem to be sufficient to postulate a direct relationship between employees’ self-transcendence and autonomy.

### Job Autonomy and Innovative Behavior

Job autonomy is known to be an important contextual antecedent of creativity and innovation (Amabile et al., 1996; Hammond et al., 2011; Anderson et al., 2014). In the meta-analysis conducted by Hammond et al. (2011), job characteristics, including job autonomy, were found to be the strongest predictors of creativity and innovation among all predictors evaluated in their study. Having freedom in performing their work, employees are able to find and develop working methods that fit them optimally (De Spiegelaere et al., 2015). Such “space” is necessary for creativity and innovative behavior because these actions are focused on experimenting and developing the best approaches to solve problems (De Spiegelaere et al., 2015). Accordingly, Dierdorff and Morgeson (2013), p. 694, argued that “by having freedom in the work role (autonomy), individuals are able to take the initiative and perform in a creative manner because they are less constrained in their role performance.”

A number of studies have confirmed that autonomy is positively related to creativity and innovation. Job autonomy was found to be positively related to employees’ innovative behavior at work (Axtell et al., 2000; Ramamoorthy et al., 2005; De Spiegelaere et al., 2014, 2015, 2016) and to job creativity (Liu et al., 2011). In line with job characteristics theory (Hackman and Oldham, 1976, 1980) and the self-determination theory (Deci et al., 1989; Ryan and Deci, 2000), which emphasize the motivational role of job autonomy, and based on the previous research findings, we postulate that job autonomy is positively related to employees’ innovative behavior.

*Hypothesis 3.* Employees’ job autonomy is positively related to their innovative behavior.

### Job Autonomy as a Mediator Between Personal Values and Innovative Behavior

**Figure 1:**
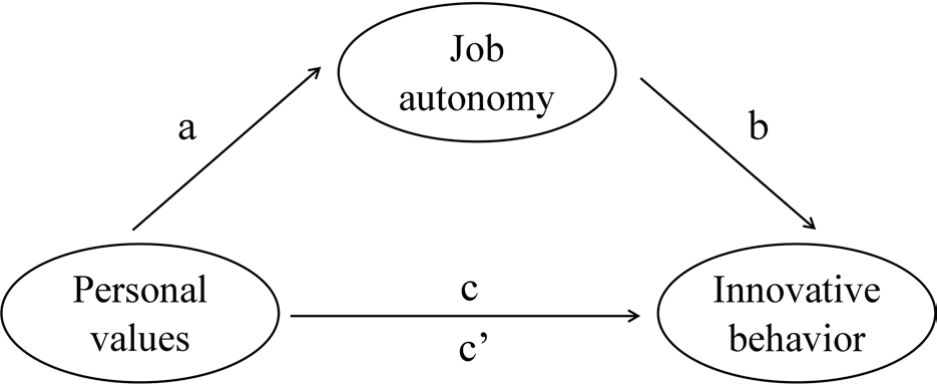
Conceptual model of the relationships tested in the study.

Although personal values have been examined as predictors of creativity and innovation in several studies (Dollinger et al., 2007; Kasof et al., 2007; Sousa and Coelho, 2011), it is still uncertain exactly how these relationships occur. For example, in a study conducted by Choi (2004), there was no confirmation of the mechanism proposing that innovative organizational culture is related to innovation-use behavior through innovative values. Therefore, it is necessary to seek other mechanisms explaining individual innovation. Hence, responding to this need, we not only postulate direct relationships between employees’ personal values and their innovative behavior but also propose that job autonomy can mediate these relationships (Figure 1).

It should be mentioned that some previous studies have treated job autonomy as a moderator, rather than a mediator, of relationships between personal values and work outcomes (e.g., Sousa et al., 2012; Sousa and Coelho, 2013). However, we propose to go beyond this pattern and verify whether job autonomy can play a mediating role. We base our expectation on an understanding of job autonomy as not only “given” by managers or derived from the job itself, as postulated by the traditional job design framework (Hackman and Oldham, 1976, 1980), but also strengthened by employees on their own, which is consistent with the proactivity conceptions (Wrzesniewski and Dutton, 2001; Berg et al., 2008; Tims and Bakker, 2010). Morgeson and Humphrey (2008) admit that managers are often responsible for designing or redesigning their subordinates’ work and frequently must even customize the work design to their employees’ individual competencies. Nevertheless, they stress that workers also play the role of proactive ‘crafters’ of their work, dynamically redesigning work to be more suitable to their specific capabilities, interests, or to the situation (Morgeson and Humphrey, 2008). We agree with this argument and expect that employees’ pursuit of job autonomy is predicted by their personal values, which are cognitive representations of their basic motivations and, therefore, affect their choices, decisions, and behaviors (Arieli and Tenne-Gazit, 2017; Sagiv and Roccas, 2017). Thus, we propose testing whether job autonomy mediates the relationship between personal values and employees’ innovative behavior.

*Hypothesis 4.* Employees’ job autonomy mediates the relationships between openness to change (*H4a*), conservation (*H4b*), self-enhancement (*H4c*), self-transcendence *(H4d*) values, and innovative behavior.

## Materials and Methods

### Procedure

Private sector employees who worked in micro-, small-, and medium-sized enterprises operating in Poland that employ 1–250 employees were invited to participate in the study. Employees working for larger companies or corporations and in the public sector were not asked to participate, as their work may be regulated by stricter company rules (Frant, 1993). The data were gathered through direct contact with the participants using a paper-and-pencil questionnaire. Participation in the study was voluntary, and the participants did not receive any reward. Confidentiality and anonymity were ensured.

### Participants

A total of 263 employees (including 138 women) participated in this study. They ranged in age from 19 to 74 years (*M* = 33.88, *SD* = 10.62). Concerning work contracts, 155 (55.6%) of the participants were employed under full-time permanent contracts, 49 (18.6%) had temporary contracts, and 59 (25.8%) had another type of work contract. Concerning education, 44.1% of the respondents had a master’s degree, 10.3% had a bachelor’s degree, 34.6% had finished high school, and only 8.7% had graduated from vocational school; six participants (2.3%) did not provide information about their education. The participants’ overall work tenure ranged from 2 months to 46 years (*M* = 11.43 years, *SD* = 10.22). Their tenure in their present company ranged from 1 month to 32 years (*M* = 5.63 years, *SD* = 6.51). The companies at which they worked employed, on average, approximately 56 people (*M* = 54.94, *SD* = 61.73, *Me* = 20.00). The participants represented seven types of occupations classified according to the International Standard Classification of Occupations (ISCO-08, 2012): professionals (36.9%), craft and related trade workers (19.8%), service workers and shop sales workers (18.3%), technicians and associate professionals (13.7%), clerks (4.9%), plant and machine operators and assemblers (3.0%), and elementary occupations (1.9%).

### Measures

#### Personal Values

To measure the personal values of the employees, the 21-item Portrait Values Questionnaire (PVQ-21) was used (Schwartz, 2003a). The measure is not cognitively challenging and is appropriate even for people with little or no formal schooling (Cieciuch, 2013; Roccas et al., 2017). The measure includes 21 statements that provide a verbal portrait of different people (i.e., their goals, aspirations, or wishes), implicitly indicating the significance of different value types (Schwartz, 2003a). Sample items include the following: *Thinking up new ideas and being creative is important to him. He likes to do things in his own original way*; *It is important to him to show his abilities. He wants people to admire what he does*. Using a 6-point scale ranging from 1 = *very much like me* to 6 = *not like me at all*, for each item, the participants answered the question, “*How much like you is this person?*” The participants’ responses were recoded so that a higher score reflected a greater importance of the value. Particular items reflect basic types of values, which can be structured into four higher-order values, namely openness to change, conservation, self-enhancement, and self-transcendence. Because hedonism is a component of both self-enhancement and openness to change values (Schwartz, 2003a), we decided to exclude it from all further analyses, following previous research (e.g., Huysentruyt et al., 2015). Schwartz (2003a) claims that people can differ in their tendencies to respond to value measures when using the response scale (e.g., some people are likely to use only one part of the response scale). Therefore, in most statistical analyses, it is critical to control for such individual differences (Schwartz, 2003b). Following this recommendation, we centered raw scores by computing each person’s mean score for all 21 items (i.e., the MRAT), and then, we calculated the corrected scores by subtracting the MRAT from the individual mean score for each value. In the current study, the internal consistency of the scale was evaluated using Cronbach’s alpha, with the following results: 0.56 for openness to change, 0.67 for conservation, 0.68 for self-enhancement, and 0.72 for self-transcendence. Such relatively low reliability results are typical for this measure because of the structure of the questionnaire (i.e., different components of the values are included in each higher-order value) and because every higher-order value was composed of a relatively small number of items (Schwartz, 2003a). Therefore, the reliability results should not be treated as a measurement weakness nor should they be considered problematic for the research results. To verify the factorial structure of the measure, confirmatory factor analysis (CFA) using AMOS software (Arbuckle, 2005; Brown, 2006) was performed. When assessing the model fit, CFI values higher than 0.90 indicate an acceptable model fit, and for the RMSEA and SRMR indices, values below 0.05 indicate a good model fit and values below 0.08 (and up to 0.10) indicate an acceptable fit; the lower the AIC index is, the better the model fits the data (Brown, 2006). According to previous recommendations (Cieciuch and Davidov, 2012), a one-factor model was tested separately for each of four higher-order values. Concerning openness to change, the factorial model showed good fit to the data (*χ*^2^_(2)_ = 0.089, *p =* 0.956, CFI = 1.00, AIC = 16.089, RMSEA = 0.000, SRMR = 0.005). In the case of conservation, the model also showed good fit to the data (*χ*^2^_(9)_ = 23.666, *p =* 0.005, CFI = 0.922, AIC = 47.666, RMSEA = 0.077, SRMR = 0.047). When testing the self-enhancement model, the analysis revealed that it had acceptable fit (*χ*^2^_(8)_ = 6.343, *p =* 0.042, CFI = 0.975, AIC = 22.343, RMSEA = 0.088, SRMR = 0.032). Finally, when we analyzed the self-transcendence model, it also showed acceptable fit to the data (*χ*^2^_(5)_ = 16.039, *p =* 0.007, CFI = 0.954, AIC = 36.039, RMSEA = 0.089, SRMR = 0.038).

#### Job Autonomy

The autonomy experienced by employees at work was measured with four items of the autonomy scale from the Work Design Questionnaire (Morgeson and Humphrey, 2006). Each item of the scale is a statement (e.g., *My job allows me to make a lot of decisions on my own*; *The job gives me a chance to use my personal initiative or judgment in carrying out the work*) to which the participants should respond using a five-point answer scale that ranges from 1 = *strongly disagree* to 5 = *strongly agree.* In the present study, Cronbach’s alpha for the autonomy scale was 0.90, which implies very good scale reliability. We conducted CFA, and the one-factor model showed good fit to the data (*χ*^2^_(1)_ = 0.249, *p =* 0.617, CFI = 1.00, AIC = 36.039, RMSEA = 0.000, SRMR = 0.003).

#### Innovative Behavior

The participants’ innovative behavior was measured using the Innovative Behavior Questionnaire (Scott and Bruce, 1994). The questionnaire includes six items (e.g., *I generate creative ideas*; *I develop adequate plans and schedules for the implementation of new ideas*). For each statement, the participants answered how frequently they behaved as described in the statement, using a five-point scale ranging from 1 = *never* to 5 = *very often*. In the present study, the scale reliability was 0.85, which indicates good internal consistency. CFA, which was conducted following Purc and Laguna’s (2017) recommendations, confirmed the one-factor structure of the measure. The present study confirmed that such a model fits well with the data (*χ*^2^_(6)_ = 7.089, *p =* 0.313, CFI = 0.998, AIC = 37.089, RMSEA = 0.026, SRMR = 0.015).

### Data Analysis Strategy

The first step was to examine whether the data suffered from the common method variance problem. Therefore, Harman’s single factor test was employed (Podsakoff et al., 2012). This technique is currently considered to be the most effective and simplest method of testing common method variance (Fuller et al., 2016). It can be applied by conducting exploratory factor analysis (Razmus and Mielniczuk, 2018). If the one-factor solution reveals that the percent of its explained variance exceeds 50–60%, then the results suffer from the common method variance (Fuller et al., 2016).

In the next step, descriptive statistics and correlations between study variables were analyzed. Previous studies have suggested that creativity and innovative behavior can be affected by sex and age (Slagter, 2009; Alsos et al., 2013; Smith et al., 2016; Hollanders and Es-Sadki, 2017). Therefore, we conducted a hierarchical multivariate regression analysis to test whether there were statistically significant effects of sex and age on innovative behavior and, consequently, whether it was necessary to control for these variables in further analyses.

The fundamental part of the data analysis was testing the postulated hypotheses. To test the hypotheses, particularly to verify whether job autonomy mediates the relationships between personal values and innovative behavior, Model 4 in the PROCESS macro (Hayes, 2013) for SPSS was applied. The use of this macro allows the estimation of an indirect effect by using a bootstrapping technique. Bias-corrected and accelerated bootstrapping with 5,000 repetitions (5,000 samples randomly generated from the whole sample) was applied in the present analyses. In terms of interpreting the results, if the confidence interval does not include zero, it indicates a statistically significant mediation (indirect) effect. The hypotheses concerning direct relationships were also verified based on the PROCESS macro results.

## Results

### Common Method Variance Test

To examine whether the data gathered in the study suffer from the common method variance, Harman’s single factor test was applied (Podsakoff et al., 2003; Fuller et al., 2016). All items of all measures used in the study were loaded into an exploratory factor analysis. An unrotated solution was used. The results showed that a single factor that accounted for most of the covariance among measures did not appear. A three-factor solution was obtained, and the first factor explained 43.93% of the variance, which did not exceed 50% of the variance explained (Fuller et al., 2016). Therefore, it was not necessary to control for common method variance in further analyses.

### Descriptive Statistics and Correlations Between the Study Variables

Descriptive statistics and Pearson’s correlations are reported in Table 1. In terms of personal values, the correlations between both bipolar dimensions are statistically significant and negative, which reflect their opposite positions on the motivational value continuum and aligns with Schwartz’s values theory (Schwartz, 1992). Openness to change values were negatively correlated with conservation (*r* = −0.69, *p* < 0.001), and self-enhancement values were negatively correlated with self-transcendence values (*r* = −0.54, *p* < 0.001).

**Table 1:** Means, standard deviations, and correlations between study variables (*N* = 263).

	Variables	*M*	*SD*	1	2	3	4	5
1	Openness to change	−0.02	0.67					
2	Conservation	0.09	0.61	−0.69^***^				
3	Self-enhancement	−0.42	0.69	0.10	−0.48^***^			
4	Self-transcendence	0.51	0.59	−0.28^***^	0.12	−0.54^***^		
5	Autonomy	3.37	0.99	0.12^*^	−0.14^*^	0.21^**^	−0.20^**^	
6	Innovative behavior	3.33	0.76	0.08	−0.22^***^	0.25^***^	−0.09	0.49^***^

*Note:* Pearson’s *r* correlations are reported. Openness to change, conservation, self-enhancement, and self-transcendence were centered.

*** *p* < 0.001;

** *p* < 0.01;

* *p* < 0.05 (two tailed).

### Control Variables

A hierarchical multivariate regression analysis was applied to test whether there was a statistically significant effect of sex and age on innovative behavior. Therefore, these two variables were entered into the model as predictors explaining innovative behavior. Sex was coded as a dichotomous variable (0 = male and 1 = female). The regression analysis results showed that neither sex nor age was significant predictors of innovative behavior (*β* = 0.04, *p* = 0.531; *β* = −0.02, *p* = 0.785, respectively). Thus, we did not control for these variables in further analyses.

### Hypotheses Testing

Mediation analyses were performed using the PROCESS macro (Model 4, Hayes, 2013). All direct and indirect effects were estimated based on these bootstrapped samples. For each of the analyses, direct effects (a, b, and c, as shown in Figure 1), an indirect effect (c′) with the bootstrapped standard error (*SEB*), and 95% confidence intervals (CIs) are reported in Table 2.

**Table 2:** Results of mediation analyses.

Predictor	Direct effects			Indirect effect		
	*a*	*b*	*c*	*c′*	*SEB*	*95% CI*
Openness to change	0.18^*^	0.37^***^	0.08	0.07	0.04	−0.01, 0.16
Conservation	−0.22^*^	0.36^*^	−0.20^**^	−0.08	0.04	−0.17, −0.01
Self-enhancement	0.30^***^	0.35^***^	0.17^**^	0.10	0.03	0.04, 0.18
Self-transcendence	−0.33^**^	0.38^***^	0.002	−0.13	0.04	−0.21, −0.05

*Note: a* = personal values – autonomy direct effect; *b* = autonomy – innovative behavior direct effect; *c* = personal values – innovative behavior direct effect; *c′* = indirect effect of autonomy. *SEB =* bootstrapped standard error; *95% CI* = 95% confidence interval. For *a*, *b*, *c* and *c′* effects, unstandardized *B* coefficients are reported.

*** *p* < 0.001;

** *p* < 0.01;

* *p* < 0.05.

First, direct effects were examined to verify hypotheses *H1a–H1c.* The analysis showed that two of the four higher-order values were related to innovative behavior. Openness to change values were not found to be a significant predictor of employee innovative behavior (*B* = 0.08, *SEB* = 0.06, *p* = 0.187). Therefore, hypothesis *H1a* is rejected. Conservation values showed a significant negative effect on innovative behavior (*B* = −0.20, *SEB* = 0.07, *p* = 0.004), which supports hypothesis *H1b*. Self-enhancement values were also revealed to be directly related to innovative behavior. As expected, the higher the importance that employees attributed to self-enhancement values, the higher their innovative behavior (*B* = 0.17, *SEB* = 0.06, *p* = 0.005). Thus, hypothesis *H1c* is supported. When investigating the relationship between self-transcendence values and innovative behavior, no statistically significant effect was detected (*B* = 0.002, *SEB* = 0.07, *p* = 0.973). We also examined whether employees’ personal values have a direct relationship with their job autonomy, which was postulated in hypotheses *H2a–H2c*. The results showed that all four higher-order values were statistically significant predictors of employees’ job autonomy. Openness to change values were found to be a positive predictor of autonomy (*B* = 0.18, *SEB* = 0.09, *p* = 0.046), which allows hypothesis *H2a* to be accepted. Conservation values were negatively related to perceived employees’ job autonomy (*B* = −0.22, *SEB* = 0.10, *p* = 0.024), confirming hypothesis *H2b*. Self-enhancement values were also shown to be a significant predictor of job autonomy, and this effect was positive (*B* = 0.30, *SEB* = 0.09, *p* < 0.001). This result indicates that hypothesis *H2c* is supported. Self-transcendence values were found to negatively predict employees’ job autonomy (*B* = −0.33, *SEB* = 0.10, *p* = 0.001). Therefore, we can conclude that there is a significant negative relationship between self-transcendence values and job autonomy in employees.

Hypothesis *H3* aimed to test the potential positive relationship between employees’ job autonomy and their innovative behavior. The examination of a direct effect of autonomy on innovative behavior confirms this hypothesis; job autonomy was a significant predictor of innovative behavior, and this relationship was positive in each of the four equations, including different personal values (for openness to change, *B* = 0.37, *SEB* = 0.04, *p* < 0.001; for conservation, *B* = 0.36, *SEB* = 0.04, *p* < 0.001; for self-enhancement, *B* = 0.35, *SEB* = 0.04, *p* < 0.001; and for self-transcendence, *B* = 0.38, *SEB* = 0.04, *p* < 0.001).

Next, indirect bootstrapped effects were analyzed to verify hypotheses *H4a–H4d*. Hypothesis *H4a* postulated that the relationship between openness to change values and innovative behavior is mediated by job autonomy. The mediation analysis showed that the indirect effect was nonsignificant (*B* = 0.07, *SEB* = 0.04, 95% CI [−0.01, 0.16]) because the 95% CI included zero. Therefore, hypothesis *H4a* is not accepted. The indirect effect of job autonomy on the relationship between conservation values and innovative behavior was found to be significant (*B* = −0.08, *SEB* = 0.04, 95% CI [−0.17, −0.01]), thereby confirming hypothesis *H4b*. Hypothesis *H4c* is supported as well – job autonomy was found to be a significant mediator of the relationship between self-enhancement values and innovative behavior (*B* = 0.10, *SEB* = 0.03, 95% CI [0.04, 0.18]). Finally, there was also a significant indirect effect of job autonomy in the relationship between self-transcendence values and innovative behavior (*B* = −0.13, *SEB* = 0.04, 95% CI [−0.21, −0.05]). Thus, this result indicates that hypothesis *H4d* is also confirmed. In summary, three of the four specific mediation hypotheses are supported. We can conclude that job autonomy is a significant mediator of the relationships between personal values (i.e., conservation, self-enhancement, and self-transcendence, but not openness to change) and employees’ innovative behavior.

## Discussion

The present study aimed to investigate the relationships between personal values, job autonomy, and innovative behavior of employees. We tested whether employees’ personal values predict their innovative behavior, on the one hand, and their job autonomy, on the other hand. We also expected that job autonomy mediates the relationships between values and innovative behavior. The study results confirmed most of our expectations.

Regarding the relationship between personal values and employees’ innovative behavior, our results revealed the effects of two of the four higher-order values, namely conservation and self-enhancement values. As expected, employees who attributed high importance to conservation values, which involve maintaining the status quo and being resistant to change, are less willing to behave innovatively at work. This result supports the postulates derived from Schwartz’s (1992) theory, which states that accepting established procedures, norms, and customary manners of behavior, which are typical for conservation values, is not conducive to innovative behavior (Schwartz and Bardi, 2001; Schwartz, 2006; Sousa et al., 2012). This result is also consistent with previous research demonstrating negative relationships between conservation values and creativity (Rice, 2006; Dollinger et al., 2007; Kasof et al., 2007; Lipponen et al., 2008; Sousa and Coelho, 2011).

Our results also showed that self-enhancement values positively predict employees’ innovative behavior. We postulated that being innovative can help to attain personal success and achieve a dominant position among other co-workers, which are the central goals of self-enhancement values (Schwartz, 1992). Employees who strongly preferred this set of higher-order values were found to be more innovative, which is consistent with previous research findings obtained by Sousa and Coelho (2011) and Taştan and Davoudi (2017). Nevertheless, our results are contradictory to those found by Dollinger et al. (2007), who found that power is negatively related to creativity. However, in their study, creativity was measured by applying methods, such as drawing creative products, developing creative stories or photo essays, which focused on the artistic aspect of creativity of university students. Therefore, the research context of this previous study differs substantially from that in the present study, in which the sample consisted of employees, and aims to investigate not creativity but innovative behavior, which is strongly grounded in the work context.

Our results also showed that there is no significant relationship between employees’ self-transcendence values and innovative behavior. This result aligns with the notion suggested by Sousa et al. (2012), who argued that self-transcendence values do not seem to be more strongly related to the work context than other higher-order values. However, Arieli and Tenne-Gazit (2017) recently proposed that universalism can be related to creativity and innovation, and other research findings showed that prosocial motivation may encourage idea development in employees (Grant and Berry, 2011). Therefore, more research concerning this issue is needed. Future research should particularly test such relationships among employees and explain not only idea generation (creativity) but also idea implementation (innovative behavior). It may also be valuable for future research to take into account prosocial motivation and include the context of social relationships in organizations.

Job autonomy was found to be predicted by all four higher-order values. As we expected, employees who attribute high importance to openness to change values (self-direction and stimulation) experience more autonomy in their work. This result aligns with Schwartz’s theory, which postulates that openness to change values are focused on independent action and thought and willingness to new experiences (Schwartz, 2003a). Our results confirmed that such motivation in employees is accompanied by striving for more autonomy in their workplace. Similarly, when employees value highly self-enhancement (power and achievement), they also experience more autonomy in their work. This result corresponds with the theory of basic human values because people who attribute high importance to self-enhancement values aim to attain success, prestige, and a dominant position over other people and demonstrate competence (Schwartz, 1992). These goals seem to be impossible to attain without having a substantial level of autonomy at work. This result is also consistent with Mumford’s arguments that power and achievement are strong motives of independent people (Mumford, 2000).

Employees’ conservation values were found to be negatively related to job autonomy, which supports our expectations [derived from Schwartz’s value theory (Schwartz, 1992)]. This result also aligns with the results obtained by Sagiv and Schwartz (2004), who demonstrated that career counseling clients who attributed high importance to conformity values behaved less independently during the counseling process. In sum, people who attribute high importance to conservation values (conformity, security and tradition), which focus on maintaining the status quo, self-restriction, and resistance to change, are not strongly predisposed to strive for job autonomy because it is potentially disturbing to established social organizational norms.

Our results also revealed that employees’ self-transcendence values are negatively related to their autonomy at work. It seems that people for whom these values are of great importance are not as focused on themselves but instead care about other people and the environment (Schwartz, 1992). They are more concentrated on pro-social and altruistic behaviors at work (Sosik et al., 2009; Schwartz, 2010) and on cooperating with others (Sagiv et al., 2011b) than on increasing their own job autonomy, which can be harmful for the autonomy of their co-workers or managers.

In summary, the results of the present study confirmed our expectations that employees’ personal values are important predictors of their job autonomy. The results seem to support the argument that jobs may be proactively redesigned by employees to be more convergent with their preferences and characteristics, such as their personal values (Wrzesniewski and Dutton, 2001; Berg et al., 2010). Nonetheless, it should be noted that, according to the traditional approach to job design (Hackman and Oldham, 1976, 1980), employee job autonomy is usually treated as a rather objective job characteristic, which depends on the nature of the job itself and on supervisors (Park and Jang, 2015). Thus, job autonomy is often considered to be a contextual moderator in explaining organizational phenomena (e.g., Molleman and van den Beukel, 2007; Sousa et al., 2012). However, we conducted an additional analysis of differences between various occupations^1^, and no significant differences in job autonomy were revealed (*F*_(6, 251)_ = 1.29, *p* = 0.261). Therefore, the level of job autonomy does not depend on the occupation type. This result supports our approach and suggests that the fact that different people have different levels of job autonomy may be a result of their own efforts; some people are motivated to strive for job autonomy (because it is congruent with their personal values), whereas others are not (when their values do not foster being autonomous). It is not only the nature of a job (assuming that some jobs are more autonomous than others) but also the personal characteristics of an employee that can shape the level of job autonomy that he/she experiences at work. This notion is in line with job crafting theory, which emphasizes that employees are proactive crafters of their work environment – their role is not reduced to working under the conditions imposed by their managers, as they can also actively shape their jobs to make them better fit their expectations and preferences (Wrzesniewski and Dutton, 2001; Berg et al., 2010). Future studies may examine behaviors that help to craft a job in terms of job autonomy to fit employees’ personal value preferences.

In the present study, we did not find a significant relationship between employees’ openness to change values and their innovative behavior. Schwartz’s (1992) theory allows us to postulate that these values are positively related to creativity and innovation. Motivational goals of openness to change (i.e., the willingness to create, choose, explore, preference for novelty, and change) seem to encourage people to behave in innovative ways. Indeed, previous research findings have shown that there are positive relationships between openness to change values and creativity (Rice, 2006; Dollinger et al., 2007; Kasof et al., 2007; Sousa and Coelho, 2011). However, in the present study, the relationship between openness to change and innovative behavior was not statistically significant. We consider several potential reasons for this result. First, some previous studies that found a positive association between openness to change values and creativity were conducted with student samples (Dollinger et al., 2007; Kasof et al., 2007). Second, because of a lack of research examining the role of individuals’ personal values on their innovative behavior, our expectations were primarily based on theoretical contributions and previous research on creativity. However, although creativity and innovative behavior are similar constructs, they are not equivalent (Anderson et al., 2014; Purc et al., 2015). Innovative behavior includes not only idea generation but also seeking support for the idea and its implementation (Scott and Bruce, 1994; Amabile, 1997; West, 2002; Anderson et al., 2004; Hammond et al., 2011), which demands cooperation with others within an organization. This again raises the issue of social relationships between managers and employees as well as among employees, which may be considered in future studies.

The present study aimed to explain the mechanism through which the personal values of employees relate to their innovative behavior. Our results revealed that job autonomy was a significant mediator of the relationships between three among four higher-order values (conservation, self-enhancement, and self-transcendence) and innovative behavior. The relationship between openness to change and innovative behavior was not mediated by job autonomy. Future research is needed to find other mechanisms through which such a relationship may occur. The mediation analysis results generally support our postulations that personal values not only motivate the pursuit of job autonomy but also are indirectly associated with innovative behavior. In addition, our study supports other findings indicating that job autonomy predicts innovative behavior (Axtell et al., 2000; Ramamoorthy et al., 2005; De Spiegelaere et al., 2014, 2015, 2016). We can conclude that employees’ personal values serve as a predisposition for functioning in the workplace and, together with other variables, such as job autonomy, relate to innovative behavior.

### Limitations

When testing the mediation mechanism, we should remember that the present study is cross-sectional, and thus, no causal conclusions can be drawn, which constitute a limitation of this study. Nevertheless, as personal values develop in childhood (Vecchione et al., 2015, 2016; Cieciuch et al., 2016), job autonomy is relatively changeable because it is dependent on the organizational context (Hackman and Oldham, 1976, 1980) and because innovative behavior based on creative ideas is performed during daily work duties (Weinberger et al., 2018), which justifies the direction of variables included in our model. However, further research concerning the relationships between employees’ personal values, job autonomy, and innovative behavior employing a longitudinal or experimental design is needed to discover the interplay between these variables over time.

In the current study, we concentrated on the role of job autonomy as a central motivational work characteristic (Morgeson and Humphrey, 2006). However, job autonomy is only one of the job features described by Hackman and Oldham’s (1976) job characteristics theory. Therefore, future studies should investigate the role of other job characteristics in relation to personal values and in stimulating innovative behavior in organizations.

Another limitation that should be addressed is that we used self-reports to measure study variables. Self-report measures seem to be the most appropriate solution to assess personal values because values are subjective motivational goals (Roccas et al., 2017). Similarly, the measurement of job autonomy seems to be necessarily subjective because the most important aspect is how an employee perceives autonomy in his/her work, not how others observe it. Objective measures of autonomy are difficult to obtain and may not refer to an employee’s actual feeling of being independent at work. Thus, self-report measures seem to be the best solution to capture perceived job autonomy. Innovative behavior was subjectively rated by employees as well, which may not reflect their actual behavior, and responses can be biased due to social desirability (Zacher et al., 2016). However, it was found that there is a significant positive correlation between the self-ratings of innovative behavior and the objective measure of invention disclosures (Scott and Bruce, 1994). In addition, Janssen (2000, 2001) found that employees’ self-ratings of innovative behavior were correlated with their supervisors’ ratings. Some researchers have also argued that employees are a good source of information about their own creativity and innovative performance (Organ and Konovsky, 1989; Janssen, 2000, 2004; Shalley et al., 2009) because it is a rather discretionary behavior, and the ratings of other people (e.g., managers or co-workers) may miss subtle, less visible innovative activities, capturing only those that are designed to make an impression. Future research should consider such problems, and researchers may use other measures.

Our study was performed in a single country, namely Poland. Because cultural differences at the societal level (Hofstede, 1980) have been considered important with respect to innovation (Rosenbusch et al., 2011), these differences may also influence the relationships between values and innovative behavior. Therefore, future cross-cultural research and/or research in other cultural contexts is encouraged.

### Practical Implications

The results of the study have some practical implications, which can be useful for managers or human resource specialists. First, it is very important to better understand the predictors of innovative behavior in organizations because innovation is one of the sources of organizational success and competitiveness (Woodman et al., 1993). Personal values are relatively stable characteristics (Schwartz, 1992), and as such, it is not easy to adapt them to specific situations. Therefore, knowing which of employees’ values are positively related to their innovative behavior, human resource departments can use this knowledge in the selection and recruitment process as well as in job design initiatives. Employing and retaining employees with high levels of self-enhancement values may increase the innovativeness of an organization. Moreover, entrepreneurs and managers may support employees’ innovative behavior by providing them with more autonomy at work and, in this way, building a more innovation-friendly job environment. It is also possible to develop innovativeness through training programmes stimulating creativity and teamwork that increase competencies to shape an environment that promotes innovation and cooperate in introducing changes (Białoń, 2010).

## Conclusions

Despite some limitations, our study offers valuable empirical evidence that allows for theory development. The results provide insight into the relationships between employees’ personal values, job autonomy, and innovative behavior, which have not been studied to date. Thus, they constitute a new perspective in innovation research, extending insights from Schwartz’s (1992) theory of basic human values to a new context. Namely, our results show that personal values can stimulate innovative behavior in the workplace. In addition, the present study investigated not only the direct relationships between personal values and innovative behavior but also the mediation mechanism. Thus, we attempted to respond to the call to reveal the mechanisms through which innovation can be driven, which was recently emphasized in the innovation literature (Hammond et al., 2011; Anderson et al., 2014, 2018). Moreover, we also addressed suggestions that personal values, as well as contextual factors, can explain behavior (Sousa et al., 2012; Arieli and Tenne-Gazit, 2017), considering job autonomy as an indicator of work context. Therefore, applying a personal values perspective to examine antecedents of job autonomy brings new insights to both basic human values theory and job design theory.

## Ethics Statement

All procedures performed in this study were in accordance with the ethical standards. Informed consent was obtained from all individual participants included in the study. Participation in the study was voluntary and the participants did not receive any reward. Respondents were asked to fill in a set of questionnaires. They were able to withdraw from the study at each moment. The confidentiality and anonymity were ensured. The study received the approval from the Ethical Committee of The John Paul II Catholic University of Lublin, Institute of Psychology.

## Author Contributions

EP and ML were involved in formulating the research question, designing the study, writing the article, and drafting and approving the final manuscript. EP was responsible for collecting and analyzing the data.

### Conflict of Interest Statement

The authors declare that the research was conducted in the absence of any commercial or financial relationships that could be construed as a potential conflict of interest.

## Footnotes

^1^Occupations were differentiated according to the International Standard Classification of Occupations (ISCO-08, 2012).

## References

[ref1] Alsos G. A. Ljunggren E. Hytti U. (2013). Gender and innovation: state of the art and a research agenda. Int. J. Gend. Entrep. 5, 236–256. 10.1108/IJGE-06-2013-0049

[ref401] Amabile T. M. (1988). A model of creativity and innovation in organizations. Res. Organ. Behav. 10, 123–167.

[ref2] Amabile T. M. (1996). Creativity in context: Update to “the social psychology of creativity”. (Boulder, CO, US: Westview Press).

[ref3] Amabile T. M. (1997). Motivating creativity in organizations: on doing what you love and loving what you do. Calif. Manag. Rev. 40, 39–58. 10.2307/41165921

[ref4] Amabile T. M. Conti R. Coon H. Lazenby J. Herron M. (1996). Assessing the work environment for creativity. Acad. Manag. J. 39, 1154–1184. 10.2307/256995

[ref5] Amos E. A. Weathington B. L. (2008). An analysis of the relation between employee-organization value congruence and employee attitudes. J. Psychol. 142, 615–631. 10.3200/JRLP.142.6.615-632, PMID: 19049240

[ref6] Anderson N. De Dreu C. K. W. Nijstad B. A. (2004). The routinization of innovation research: a constructively critical review of the state-of-the-science. J. Organ. Behav. 25, 147–173. 10.1002/job.236

[ref7] Anderson N. Potočnik K. Bledow R. Hülsheger U. R. Rosing K. (2018). “Innovation and creativity in organizations” in The SAGE Handbook of Industrial, Work and Organizational Psychology. Vol. 3 Second edn. eds. Ones D. S. Anderson N. Sinangil H. K. Viswesvaran C. (London: SAGE Publications). 10.4135/9781473914964

[ref8] Anderson N. Potočnik K. Zhou J. (2014). Innovation and creativity in organizations: a state-of-the-science review, prospective commentary, and guiding framework. J. Manag. 40, 1297–1333. 10.1177/0149206314527128

[ref9] Arbuckle J. (2005). Amos 6.0 user’s guide. (Spring House, PA: Amos Development Corporation).

[ref10] Arieli S. Tenne-Gazit O. (2017). “Values and behavior in a work environment: taking a multi-level perspective” in Values and behavior. Taking a cross-cultural perspective. eds. Roccas S. Sagiv L. (Berlin: Springer), 115–141.

[ref11] Axtell C. M. Holman D. J. Unsworth K. L. Wall T. D. Waterson P. E. (2000). Shopfloor innovation: facilitating the suggestion and implementation of ideas. J. Occup. Organ. Psychol. 73, 265–385. 10.1348/096317900167029

[ref12] Bardi A. Schwartz S. H. (2003). Values and behavior: strength and structure of relations. Personal. Soc. Psychol. Bull. 29, 1207–1220. 10.1177/0146167203254602 15189583

[ref13] Barron F. (1997). “Introduction” in Creators on creating: Awakening and cultivating the imaginative mind. eds. Barron F. Montuori A. Barron A. (New York: Tarcher Perigee), 1–21.

[ref14] Berg J. M. Dutton J. E. Wrzesniewski A. (2008). What is job crafting and why does it matter? (Ann Arbor, MI: University of Michigan Ross School of Business). Available at: http://www.bus.umich.edu/Positive/POS-Teaching-and-Learning/ListPOS-Cases.htm [Access: 9 April 2019].

[ref15] Berg J. M. Grant A. M. Johnson V. (2010). When callings are calling: crafting work and leisure in pursuit of unanswered occupational callings. Organ. Sci. 21, 973–994. 10.1287/orsc.1090.0497

[ref16] Białoń L. (2010). Zarządzanie działalnością innowacyjną. 1st edn. (Warszawa: Wydawnictwo PLACET).

[ref17] Biron M. Bamberger P. (2010). The impact of structural empowerment on individual well-being and performance: taking agent preferences, self-efficacy and operational constraints into account. Hum. Relat. 63, 163–191. 10.1177/0018726709337039

[ref18] Brown T. A. (2006). Confirmatory factor analysis for applied research. (New York: Guilford Press).

[ref19] Camisón-Zornoza C. Lapiedra-Alcamí R. Segarra-Ciprés M. Boronat-Navarro M. (2004). A meta-analysis of innovation and organizational size. Organ. Stud. 25, 331–361. 10.1177/0170840604040039

[ref20] Choi J. N. (2004). Individual and contextual dynamics of innovation-use behavior in organizations. Hum. Perform. 17, 397–414. 10.1207/s15327043hup1704_3

[ref21] Cieciuch J. (2013). Kształtowanie się systemu wartości od dzieciństwa do wczesnej dorosłości. (Warszawa: Wydawnictwo Liberi Libri).

[ref22] Cieciuch J. (2017). “Exploring the complicated relationship between values and behaviour” in Values and behavior: Taking a cross-cultural perspective. eds. Roccas S. Sagiv L. (Berlin: Springer), 237–248.

[ref23] Cieciuch J. Davidov E. (2012). A comparison of the invariance properties of the PVQ-40 and the PVQ-21 to measure human values across German and Polish samples. Surv. Res. Method. 6, 37–48. 10.18148/srm/2012.v6i1.5091

[ref24] Cieciuch J. Davidov E. Algesheimer R. (2016). The stability and change of value structure and priorities in childhood: a longitudinal study. Soc. Dev. 25, 503–527. 10.1111/sode.12147

[ref25] Damanpour F. (1991). Organizational innovation: a meta-analysis of effects of determinants and moderators. Acad. Manag. J. 34, 555–590. 10.2307/256406

[ref26] de Jong J. P. J. den Hartog D. (2010). Measuring innovative work behaviour. Creat. Innov. Manag. 19, 23–36. 10.1111/j.1467-8691.2010.00547.x

[ref27] De Spiegelaere S. Gyes G. Hootegem G. (2016). Not all autonomy is the same. Different dimensions of job autonomy and their relation to work engagement and innovative work behavior. Hum. Factor. Ergon. Manuf. Serv. Ind. 26, 515–527. 10.1002/hfm.20666

[ref28] De Spiegelaere S. Van Gyes G. De Witte H. Niesen W. Van Hootegem G. (2014). On the relation of job insecurity, job autonomy, innovative work behaviour and the mediating effect of work engagement. Creat. Innov. Manag. 23, 318–330. 10.1111/caim.12079

[ref29] De Spiegelaere S. Van Gyes G. De Witte H. Van Hootegem G. (2015). Job design, work engagement and innovative work behavior: a multi-level study on Karasek’s learning hypothesis. Manag. Rev. 26, 123–137. 10.1688/mrev-2015-02-DeSpiegelaere

[ref30] Deci E. L. Connell J. P. Ryan R. M. (1989). Self-determination in a work organization. J. Appl. Psychol. 74, 580–590. 10.1037/0021-9010.74.4.580

[ref31] Deci E. L. Ryan R. M. (2000). The ‘what’ and ‘why’ of goal pursuits: human needs and the self-determination of behavior. Psychol. Inq. 11:227. 10.1207/S15327965PLI1104_01

[ref32] Dierdorff E. C. Morgeson F. P. (2013). Getting what the occupation gives: exploring multilevel links between work design and occupational values. Pers. Psychol. 66, 687–721. 10.1111/peps.12023

[ref33] Dollinger S. J. Burke P. A. Gump N. W. (2007). Creativity and values. Creat. Res. J. 19, 91–103. 10.1080/10400410701395028

[ref34] Feather N. T. (1988). From values to actions: recent applications of the expectancy-value model. Aust. J. Psychol. 40, 105–124. 10.1080/00049538808259076

[ref402] Frant H. (1993). Rules and governance in the public sector: The case of civil service. Am. J. Pol. Sci. 37, 990–1007. 10.2307/2111540

[ref35] Fuller C. M. Simmering M. J. Atinc G. Atinc Y. Babin B. J. (2016). Common methods variance detection in business research. J. Bus. Res. 69, 3192–3198. 10.1016/j.jbusres.2015.12.008

[ref36] Gorgievski M. J. Stephan U. Laguna M. Moriano J. A. (2017). Predicting entrepreneurial career intentions: values and the theory of planned behavior. J. Career Assess. 26, 457–475. 10.1177/1069072717714541 30443149 PMC6196350

[ref403] Grant A. M. Berry J. W. (2011). The necessity of others is the mother of invention: intrinsic and prosocial motivations, perspective taking, and creativity. Acad. Manag. J. 54, 73–96.

[ref37] Gump N. W. (2007). Creativity and self knowledge: Predicting creativity with values and vocational interests measures. (US: ProQuest Information & Learning).

[ref38] Hackman J. R. Oldham G. R. (1976). Motivation through the design of work: test of a theory. Organ. Behav. Hum. Perform. 16, 250–279. 10.1016/0030-5073(76)90016-7

[ref39] Hackman J. R. Oldham G. R. (1980). Work redesign. (Reading, MA: Addison-Wesley).

[ref40] Halbesleben J. R. B. (2010). “A meta-analysis of work engagement: relationships with burnout, demands, resources, and consequences” in Work engagement: A handbook of essential theory and research. ed. Bakker A. B. (New York, NY, US: Psychology Press), 102–117.

[ref41] Hammond M. M. Neff N. L. Farr J. L. Schwall A. R. Zhao X. (2011). Predictors of individual-level innovation at work: a meta-analysis. Psychol. Aesthet. Creat. Arts 5, 90–105. 10.1037/a0018556

[ref42] Hayes A. F. (2013). Introduction to mediation, moderation, and conditional process analysis: A regression-based approach (1 edition). (New York: The Guilford Press).

[ref43] Helson R. (1990). “Creativity in women: outer and inner views over time” in Sage focus editions. eds. Runco M. A. Albert R. S., Vol. 115. Theories of creativity (Thousand Oaks, CA, US: SAGE Publications), 46–58.

[ref44] Helson R. (1996). In search of the creative personality. Creat. Res. J. 9, 295–306. 10.1207/s15326934crj0904_1

[ref450] Hofstede G. (1980). Culture’s consequences: International differences in work-related values. (Beverly Hills, CA: Sage).

[ref45] Holland J. L. (1997). Making vocational choices: A theory of vocational personalities and work environments (Subsequent edition). (Odessa, Florida: Psychological Assessment Resources).

[ref46] Hollanders H. Es-Sadki N. (2017). European Innovation Scoreboard 2017. (Luxembourg: European Comission).

[ref48] Humphrey S. E. Nahrgang J. D. Morgeson F. P. (2007). Integrating motivational, social, and contextual work design features: a meta-analytic summary and theoretical extension of the work design literature. J. Appl. Psychol. 92, 1332–1356. 10.1037/0021-9010.92.5.1332, PMID: 17845089

[ref49] Huysentruyt M. Stephan U. Vujić S. (2015 March). CEO’s values, management style and firm performance: Evidence from social enterprise in Europe. Available at: http://chaire-eppp.org/files_chaire/huysentruyt_stephan_and_vujic_march_2015.pdf [Access: 9 April 2019].

[ref50] ISCO-08 (2012). International standard classification of occupations. Volume 1: Structure, group definitions and correspondence tables. (Geneva: International Labour Office). Available at: http://www.ilo.org/public/english/bureau/stat/isco/docs/publication08.pdf [Access: 9 April 2019].

[ref51] Janssen O. (2000). Job demands, perceptions of effort-reward fairness and innovative work behaviour. J. Occup. Organ. Psychol. 73, 287–302. 10.1348/096317900167038

[ref52] Janssen O. (2001). Fairness perceptions as a moderator in the curvilinear relationships between job demands, and job performance and job satisfaction. Acad. Manag. J. 44, 1039–1050. 10.2307/3069447

[ref53] Janssen O. (2004). How fairness perceptions make innovative behavior more or less stressful. J. Organ. Behav. 25, 201–215. 10.1002/job.238

[ref54] Janssen O. van de Vliert E. West M. (2004). The bright and dark sides of individual and group innovation: a special issue introduction. J. Organ. Behav. 25, 129–145. 10.1002/job.242

[ref55] Kasof J. Chen C. Himsel A. Greenberger E. (2007). Values and creativity. Creat. Res. J. 19, 105–122. 10.1080/10400410701397164

[ref56] Knafo A. Sagiv L. (2004). Values and work environment: mapping 32 occupations. Eur. J. Psychol. Educ. 19, 255–273. 10.1007/BF03173223

[ref57] Lipponen J. Bardi A. Haapamäki J. (2008). The interaction between values and organizational identification in predicting suggestion-making at work. J. Occup. Organ. Psychol. 81, 241–248. 10.1348/096317907X216658

[ref58] Liu D. Chen X.-P. Yao X. (2011). From autonomy to creativity: a multilevel investigation of the mediating role of harmonious passion. J. Appl. Psychol. 96, 294–309. 10.1037/a0021294, PMID: 21058804

[ref59] Meglino B. M. Ravlin E. C. (1998). Individual values in organizations: concepts, controversies, and research. J. Manag. 24, 351–389. 10.1016/S0149-2063(99)80065-8

[ref60] Molleman E. van den Beukel A. (2007). Worker flexibility and its perceived contribution to performance: The moderating role of task characteristics. Hum. Factors Man. 17, 117–135. 10.1002/hfm.20069

[ref61] Morgeson F. P. Humphrey S. E. (2006). The Work Design Questionnaire (WDQ): developing and validating a comprehensive measure for assessing job design and the nature of work. J. Appl. Psychol. 91, 1321–1339. 10.1037/0021-9010.91.6.1321, PMID: 17100487

[ref47] Morgeson F. P. Humphrey S. E. (2008). “Job and team design: toward a more integrative conceptualization of work design” in Research in personnel and human resources management. ed. Martocchio J. J., Vol. 27 (Emerald Group Publishing Limited), 39–91.

[ref62] Mumford M. D. (2000). Managing creative people: strategies and tactics for innovation. Hum. Resour. Manag. Rev. 10, 313–351. 10.1016/S1053-4822(99)00043-1

[ref63] Organ D. W. Konovsky M. (1989). Cognitive versus affective determinants of organizational citizenship behavior. J. Appl. Psychol. 74, 157–164. 10.1037/0021-9010.74.1.157

[ref630] Orth M. Volmer J. (2017). Daily within-person effects of job autonomy and work engagement on innovative behaviour: The cross-level moderating role of creative self-efficacy. Eur. J. Work Organ. Psychol. 26, 601–612. 10.1080/1359432X.2017.1332042

[ref64] Park R. Jang S. J. (2015). Mediating role of perceived supervisor support in the relationship between job autonomy and mental health: moderating role of value–means fit. Int. J. Hum. Resour. Manag. 28, 703–723. 10.1080/09585192.2015.1109536

[ref65] Podsakoff P. M. MacKenzie S. B. Lee J.-Y. Podsakoff N. P. (2003). Common method biases in behavioral research: a critical review of the literature and recommended remedies. J. Appl. Psychol. 88:879. 10.1037/0021-9010.88.5.879, PMID: 14516251

[ref66] Podsakoff P. M. MacKenzie S. B. Podsakoff N. P. (2012). Sources of method bias in social science research and recommendations on how to control it. Annu. Rev. Psychol. 63, 539–569. 10.1146/annurev-psych-120710-100452, PMID: 21838546

[ref67] Purc E. Laguna M. (2017). Factorial structure and measurement invariance of the Innovative Behavior Questionnaire. J. Creat. Behav. 1–7. 10.1002/jocb.215

[ref68] Purc E. Wałachowska K. Żaliński A. Mielniczuk E. Patynowska E. Łaguna M. (2015). Innowacja w organizacji: Sposoby ujmowania i przegląd uwarunkowań. Zag. Naukozn. 4, 425–445.

[ref69] Ramamoorthy N. Flood P. C. Slattery T. Sardessai R. (2005). Determinants of innovative work behaviour: development and test of an integrated model. Creat. Innov. Manag. 14, 142–150. 10.1111/j.1467-8691.2005.00334.x

[ref70] Razmus W. Laguna M. (2018). Dimensions of entrepreneurial success: a multilevel study on stakeholders of micro-enterprises. Front. Psychol. 9:791. 10.3389/fpsyg.2018.00791, PMID: 29892242 PMC5985317

[ref71] Razmus W. Mielniczuk E. (2018). Błąd wspólnej metody w badaniach kwestionariuszowych. Pol. Forum Psychol. 23, 277–290. 10.14656/PFP20180204

[ref72] Retowski S. Podsiadły M. J. (2016). Gdy nasza praca pasuje do naszych wartości. Ocena zgodności wartości własnych i organizacji a wypalenie zawodowe. Psychol. Społecz. 11, 56–68. 10.7366/1896180020163605

[ref73] Rice G. (2006). Individual values, organizational context, and self-perceptions of employee creativity: evidence from Egyptian organizations. J. Bus. Res. 59, 233–241. 10.1016/j.jbusres.2005.08.001

[ref74] Roccas S. Sagiv L. Navon M. (2017). “Methodological issues in studying personal values” in Values and behavior: Taking a cross-cultural perspective. eds. Roccas S. Sagiv L. (Berlin: Springer), 15–50.

[ref75] Rokeach M. (1973). The nature of human values. (New York: Free Press).

[ref76] Ros M. Schwartz S. H. Surkiss S. (1999). Basic individual values, work values, and the meaning of work. Appl. Psychol. 48, 49–71. 10.1111/j.1464-0597.1999.tb00048.x

[ref77] Rosenbusch N. Brinckmann J. Bausch A. (2011). Is innovation always beneficial? A meta-analysis of the relationship between innovation and performance in SMEs. J. Bus. Ventur. 26, 441–457. 10.1016/j.jbusvent.2009.12.002

[ref78] Ryan R. M. Deci E. L. (2000). Self-determination theory and the facilitation of intrinsic motivation, social development, and well-being. Am. Psychol. 55, 68–78. 10.1037/0003-066X.55.1.68, PMID: 11392867

[ref79] Sagiv L. (2002). Vocational interests and basic values. J. Career Assess. 10, 233–257. 10.1177/1069072702010002007

[ref80] Sagiv L. Roccas S. (2017). “What personal values are and what they are not: taking a cross-cultural perspective” in Values and behavior: Taking a cross cultural perspective. eds. Roccas S. Sagiv L. (Berlin: Springer), 3–13.

[ref81] Sagiv L. Schwartz S. H. (2000). Value priorities and subjective well-being: direct relations and congruity effects. Eur. J. Soc. Psychol. 30, 177–198. 10.1002/(SICI)1099-0992(200003/04)30:2<177::AID-EJSP982>3.0.CO;2-Z

[ref82] Sagiv L. Schwartz S. H. (2004). Values, intelligence and client behavior in career counseling: a field study. Eur. J Psychol. Educ.-EJPE 19, 237–254. 10.1007/BF03173222

[ref83] Sagiv L. Schwartz S. H. Arieli S. (2011a). “Personal values, national culture and organizations: insights applying the Schwartz value framework” in The handbook of organizational culture and climate. Second edn. eds. Ashkanasy N. N. Wilderom C. Peterson M. F. (Newbury Park, CA: SAGE), 515–537.

[ref84] Sagiv L. Sverdlik N. Schwarz N. (2011b). To compete or to cooperate? Values’ impact on perception and action in social dilemma games. Eur. J. Soc. Psychol. 41, 64–77. 10.1002/ejsp.729

[ref85] Sarros J. C. Tanewski G. A. Winter R. P. Santora J. C. Densten I. L. (2002). Work alienation and organizational leadership. Br. J. Manag. 13, 285–304. 10.1111/1467-8551.00247

[ref86] Schwartz S. H. (1992). “Universals in the content and structure of values: theoretical advances and empirical tests in 20 countries” in Advances in experimental social psychology. ed. Zanna M. P., Vol. 25 (San Diego, CA, US: Academic Press), 1–65.

[ref87] Schwartz S. H. (1994). Are there universal aspects in the structure and contents of human values? J. Soc. Issues 50, 19–45. 10.1111/j.1540-4560.1994.tb01196.x

[ref88] Schwartz S. H. (2003a). A proposal for measuring value orientations across nations. Questionnaire Development Report of ESS, 259–290.

[ref89] Schwartz S. H. (2003b). Computing scores for the 10 human values (European Social Survey). (The Hebrew University of Jerusalem). Available at: http://ess.nsd.uib.no/ess/doc/ess1_human_values_scale.pdf [Access: 9 April 2019].

[ref90] Schwartz S. H. (2006). Basic human values: theory, measurement, and applications. Rev. Fr. Sociol. 47, 929–968. 10.3917/rfs.474.0929

[ref91] Schwartz S. H. (2010). “Basic values: how they motivate and inhibit prosocial behavior” in Prosocial motives, emotions, and behavior: The better angels of our nature. eds. Mikulincer M. Shaver P. R. Mikulincer M. Shaver P. R. (Washington, DC, US: American Psychological Association), 221–241.

[ref92] Schwartz S. H. Bardi A. (2001). Value hierarchies across cultures: taking a similarities perspective. J. Cross-Cult. Psychol. 32, 268–290. 10.1177/0022022101032003002

[ref94] Scott S. G. Bruce R. A. (1994). Determinants of innovative behavior: a path model of individual innovation in the workplace. Acad. Manag. J. 37, 580–607. 10.2307/256701

[ref95] Shalley C. E. Gilson L. L. Blum T. C. (2009). Interactive effects of growth need strength, work context, and job complexity on self-reported creative performance. Acad. Manag. J. 52, 489–505. 10.5465/AMJ.2009.41330806

[ref96] Slagter F. (2009). HR practices as predictors for knowledge sharing and innovative behaviour: a focus on age. Intern. J. Hum. Resour. Dev. Manag. 9, 223–249. 10.1504/IJHRDM.2009.023454

[ref97] Smith R. M. Sardeshmukh S. R. Combs G. M. (2016). Understanding gender, creativity, and entrepreneurial intentions. Educ. Train. 58, 263–282. 10.1108/ET-06-2015-0044

[ref98] Sosik J. J. Jung D. Dinger S. L. (2009). Values in authentic action: examining the roots and rewards of altruistic leadership. Group Org. Manag. 34, 395–431. 10.1177/1059601108329212

[ref99] Sousa C. M. P. Coelho F. (2011). From personal values to creativity: evidence from frontline service employees. Eur. J. Mark. 45, 1029–1050. 10.1108/03090561111137598

[ref100] Sousa C. M. P. Coelho F. (2013). Exploring the relationship between individual values and the customer orientation of front-line employees. J. Mark. Manag. 29, 1653–1679. 10.1080/0267257X.2013.798674

[ref101] Sousa C. M. P. Coelho F. Guillamon-Saorin E. (2012). Personal values, autonomy, and self-efficacy: evidence from frontline service employees. Int. J. Sel. Assess. 20, 159–170. 10.1111/j.1468-2389.2012.00589.x

[ref102] Taştan D. D. S. Davoudi S. M. M. (2017). The relationship between organisational climate and organisational innovativeness: testing the moderating effect of individual values of power and achievement. Internat. J. Bus. Innov. Res. 12, 465–483. 10.1504/IJBIR.2017.10003335

[ref103] Tims M. Bakker A. B. (2010). Job crafting: towards a new model of individual job redesign. SAJIP: S. Afr. J. Ind. Psychol. 36, 12–20. 10.4102/sajip.v36i2.841

[ref104] Tims M. Bakker A. B. Derks D. (2012). Development and validation of the job crafting scale. J. Vocat. Behav. 80, 173–186. 10.1016/j.jvb.2011.05.009

[ref105] Tims M. Bakker A. B. Derks D. (2013). The impact of job crafting on job demands, job resources, and well-being. J. Occup. Health Psychol. 18, 230–240. 10.1037/a0032141, PMID: 23506549

[ref106] Vecchione M. Döring A. K. Alessandri G. Marsicano G. Bardi A. (2015). Reciprocal relations across time between basic values and value-expressive behaviors: a longitudinal study among children. Soc. Dev. 25, 528–547. 10.1111/sode.12152

[ref107] Vecchione M. Schwartz S. H. Alessandri G. Döring A. K. Castellani V. Caprara M. G. (2016). Stability and change of basic personal values in early adulthood: an 8-year longitudinal study. J. Res. Pers. 63, 111–122. 10.1016/j.jrp.2016.06.002

[ref108] Verhees F. J. H. M. Meulenberg M. T. G. (2004). Market orientation, innovativeness, product innovation, and performance in small firms. J. Small Bus. Manag. 42, 134–154. 10.1111/j.1540-627X.2004.00102.x

[ref109] Weinberger E. Wach D. Stephan U. Wegge J. (2018). Having a creative day: understanding entrepreneurs’ daily idea generation through a recovery lens. J. Bus. Ventur. 33, 1–19. 10.1016/j.jbusvent.2017.09.001

[ref110] West M. A. (2002). Sparkling fountains or stagnant ponds: an integrative model of creativity and innovation implementation in work groups. Appl. Psychol. Int. Rev. 51, 355–387. 10.1111/1464-0597.00951

[ref111] West M. A., Farr J. L., (Eds.) (1992). “Innovation at work” in Innovation and creativity at work: Psychological and organizational strategies. (Chichester, England, New York: Wiley).

[ref112] Woodman R. W. Sawyer J. E. Griffin R. W. (1993). Toward a theory of organizational creativity. Acad. Manag. Rev. 18, 293–321. 10.5465/AMR.1993.3997517

[ref113] Wrzesniewski A. Dutton J. E. (2001). Crafting a job: revisioning employees as active crafters of their work. Acad. Manag. Rev. 26, 179–201. 10.5465/AMR.2001.4378011

[ref114] Yuan F. Woodman R. W. (2010). Innovative behavior in the workplace: the role of performance and image outcome expectations. Acad. Manag. J. 53, 323–342. 10.5465/AMJ.2010.49388995

[ref115] Zacher H. Robinson A. J. Rosing K. (2016). Ambidextrous leadership and employees’ self-reported innovative performance: the role of exploration and exploitation behaviors. J. Creat. Behav. 50, 24–46. 10.1002/jocb.66

